# Laparoscopic Management of a Trapped Placenta Following Normal Delivery in a Woman With a Mullerian Anomaly

**DOI:** 10.7759/cureus.64051

**Published:** 2024-07-08

**Authors:** Baris Kaya, Akin Varlik, Ayse O Savkli, Berna Aslan Çetin, İbrahim Polat

**Affiliations:** 1 Obstetrics and Gynecology, Basaksehir Cam and Sakura City Hospital, Istanbul, TUR

**Keywords:** manual removal of placenta, postpartum haemorrgage, laparoscopy, mullerian anomaly, retained placenta

## Abstract

Entrapped placenta following vaginal delivery is an uncommon complication. In resistant cases, it needs to be removed by laparotomy, although this is exceptionally rare. Here, we report a 28-year-old woman, 33 weeks pregnant through in vitro fertilization, who delivered a premature male baby weighing 2400 grams with an Apgar score of 7. After delivery, the placenta remained in the unicornuate uterus. Ultrasound ruled out placenta accreta spectrum, and manual removal attempts under anesthesia failed due to lower uterine segment contraction despite using nitroglycerine. Conservative management with misoprostol and broad-spectrum antibiotics was initiated. However, increasing C-reactive protein levels and abdominal pain necessitated a computerized tomography scan, revealing the placenta trapped in the unicornuate uterus. Thirty-six hours after the delivery, the decision was made to remove the placenta laparoscopically instead of laparotomy. A unicornuate uterus containing a placenta on the right and the left rudimentary horn connected to the right uterus with bilateral adnexa, including theca cysts, were revealed during laparoscopic observation. No pelvic organ injury was noted. The placenta was removed via a fundal incision with a monopolar hook and using claw traumatic forceps. The uterus was closed with V-lock sutures; additional Z-sutures were applied. A 270-gram entire placenta was extracted using an endo bag successfully. The patient was discharged several days after the procedure without any complications. Laparoscopic extraction of a third-trimester placenta can successfully be used in resistant cases while avoiding laparotomy, even in the early postpartum period.

## Introduction

Retained placenta following vaginal delivery is a rare complication that is defined as failure to expel the placenta within 30 minutes of the neonate's birth [1]. Retained placenta can be a life-threatening situation, ending up with endometritis and postpartum hemorrhage [2-4]. To date, the management of a retained placenta was manual or instrument extraction of the placenta vaginally under anesthesia, and if all failed, then laparotomy was indicated [3,5]. In contrast to the traditional obstetric approach, here, we report a minimally invasive method, laparoscopic extraction, for dealing with a trapped placenta after vaginal delivery in a woman with a unicornuate uterus for the first time in the medical literature.

## Case presentation

**Figure 1:**
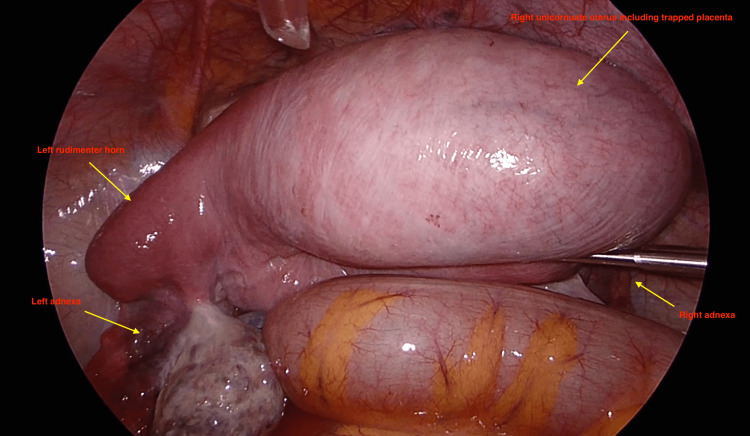
Laparoscopic view of the right unicornuate uterus including the entrapped placenta left rudimentary horn and both adnexa

A 28-year-old woman who was 33 weeks pregnant (conceived through in vitro fertilization) gave birth to a male baby weighing 2400 grams. The baby had an Apgar score of 7 and was delivered prematurely after the rupture of membranes for three days. The third stage of labor was managed with oxytocin and methylergonovine. Thirty minutes after the delivery, the placenta was found on the fundus of the unicorn uterus and did not come out on its own. Immediately, an ultrasound was performed, and the entire placenta was revealed on the fundus of the uterus, with no signs of myometrial invasion. After waiting for a spontaneous expulsion of the placenta for 1.5 hours, an attempt was made to remove the placenta manually under general anesthesia with the help of transabdominal ultrasound guidance. Nevertheless, the placenta could not be removed manually or with instruments due to lower uterine segment contraction. Therefore, a decision was made for medical conservative management using misoprostol and broad-spectrum antibiotic therapy. However, there was no indication of placental separation. Still, there were increasing levels of C-reactive protein (CRP) (postpartum 6-hour CRP 45 mg/dl, postpartum 36-hour CRP 118 mg/dl) and increased abdominal pain and distension. A computerized tomography (CT) scan was done to check for any pelvic organ injury during vaginal procedures with instruments. The CT scan was reported as a 19*4 cm hyperdense mass with gas bubbles inside the uterine myometrium, with no signs of pelvic organ injury (see Figure 1). The patient was complaining of abdominal pain and lethargy. After receiving blood and blood products due to postpartum anemia (hemoglobin dropped from 12.4 to 7.4 g/dL after delivery), the patient was still feeling weak, and pelvic pain was persisting. To prevent further deterioration of the patient’s condition, a second attempt at manual removal of the placenta was planned 36 hours after delivery under laparoscopic view. 

**Figure 2:**
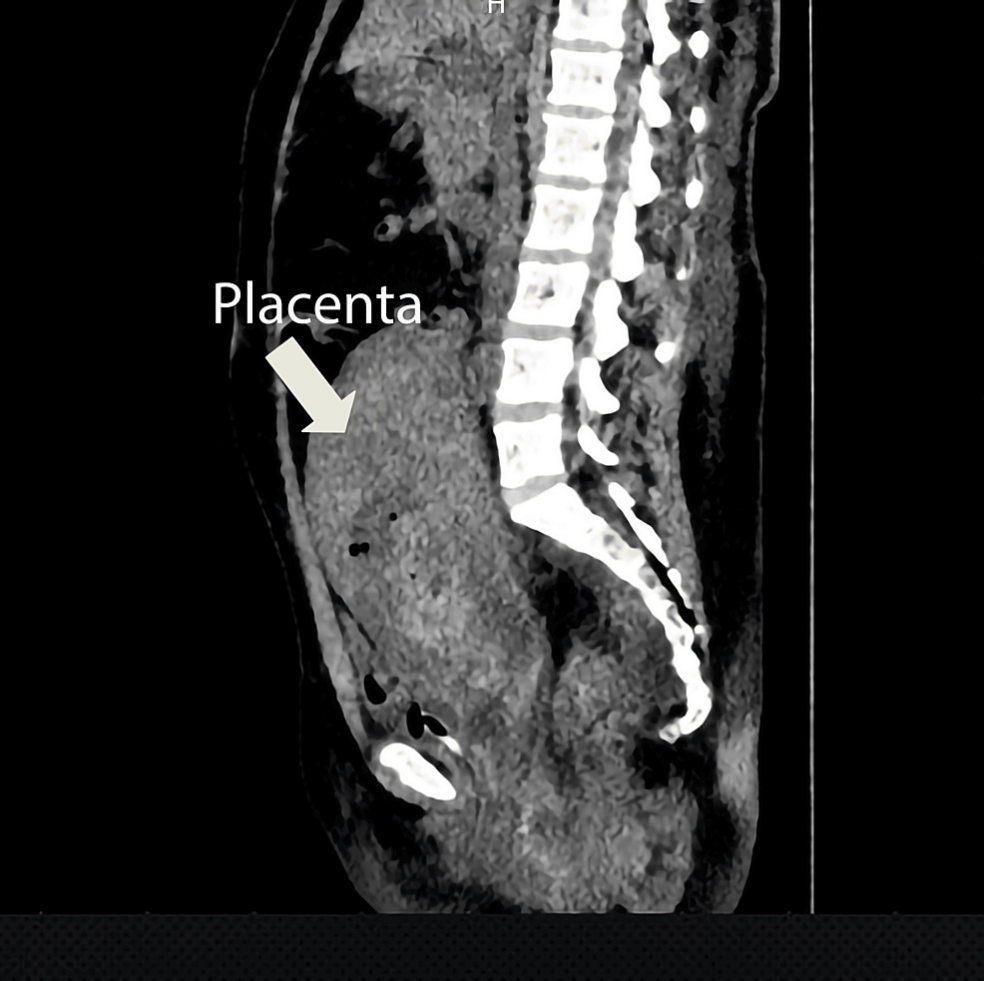
A sagittal computerized tomography (CT) view of the uterus, including the entrapped placenta

A postpartum unicornuate uterus, including the placenta extending to the umbilicus, was observed during the laparoscopy. The right unicornuate uterus was connected to a left rudimentary horn, accompanied by bilateral adnexa, including theca cysts, as seen in Figure 2 and Video 1. Additionally, no injury to the pelvic organs has been detected from previous attempts. To relax the uterus, the patient was given 100 micrograms of intravenous nitroglycerine. However, due to the strict lower uterine segment, instruments could not reach the placenta vaginally again.

**Video 1:** A narrative video demonstration of laparoscopic management of the entrapped placenta following delivery in a woman with a unicornuate uterus

It was decided to remove the placenta laparoscopically, which was already in place. The right unicorn uterine fundus, where the placenta was bulging, was cut transversally with the monopolar hook for about 8-10 cm under 20 IU oxytocin infusion. The trapped placenta was removed entirely from the uterine cavity using two 10 mm claw traumatic forceps. There was no placenta accreta. The fundal transverse incision was closed with a 0 V-lock barbed suture. After additional z-sutures to strengthen the incision and bipolar coagulation to control oozing bleeding, the placenta, weighing 270 grams, was removed from the abdomen with a 10mm endo bag through a slightly enlarged fascia and skin opening. The blood and blood clots inside the abdomen were removed by aspiration, followed by sterile suction irrigation using 1000 liters of sterile saline solution. A 10 mm Jackson-Pratt drain was placed from a 5 mm trocar. All trocar sites, including the 10 mm trocar site fascias, were closed primarily. The patient recovered quickly and was discharged without any complications several days later.

## Discussion

Our case demonstrates that laparoscopic extraction can effectively be utilized to remove a retained or trapped placenta following vaginal delivery without the need for laparotomy. These types of placental retention or entrapment are uncommon and often occur in cases of congenital uterine anomalies, which can lead to a constricted cervix or lower segment of the uterus [3]. Congenital uterine anomalies, also known as Mullerian anomalies, result from abnormalities in the formation, fusion, or resorption of the Mullerian duct during embryogenesis [6]. The percentage of Müllerian anomalies associated with pregnancy was reported as 0.40% [7]. Müllerian anomalies are divided into specific groups according to the anatomic relevance of the absence/presence of uterine corpus and cervix or the anomaly of uterus cervix and vagina. In a unicornuate uterus, one uterine cavity is usually normal, with a fallopian tube, cervix, and a rudimentary horn or anlage (communicate or non-communicate with the uterus) [6].

Studies have indicated that pregnancies involving Mullerian anomalies, including unicornuate uterus, are associated with higher rates of obstetric adverse outcomes, such as abortions, preterm labor, premature rupture of membranes, intrauterine growth restriction, placental abnormalities (placenta previa, accreta), and retained placenta as in our specific case [7-9]. Medical treatments and vaginal attempts may prove unsuccessful in these situations. Invitro fertilization conception and using methylergonovine as a uterotonic agent in the active management of the third stage of labor have also been shown to cause higher rates of the retained placenta following vaginal delivery [10,11].

In the literature, a trapped placenta following vaginal delivery is traditionally managed through either laparotomy and hysterotomy, laparotomy and hysterectomy (including the placenta), or leaving the placenta in situ and waiting for reabsorption [2-5]. In a case, laparotomy was used to extract the trapped placenta following the vaginal delivery in a 35-week pregnant woman [5]. Similarly, multiple vaginal attempts failed; therefore, laparotomy and hysterotomy were performed to extract a trapped placenta in a septate uterus. Another trapped placenta with a Mullerian anomaly was reported in a bicornuate uterus of a woman who gave birth at 37 weeks of gestation [2]. Multiple attempts to remove the placenta manually were unsuccessful, and a median uterine incision was performed to extract it (323 grams). During the laparotomy, the estimated blood loss was 4000 ml, which was significantly higher than our case (500 ml).

Leaving the placenta in situ for reabsorption is another option; however, it needs close monitoring and can result in sepsis and unexpected hemorrhage [2]. In our case, due to rising CRP levels and persistent abdominal pain, a surgical method was necessary to remove the placenta immediately, avoiding the possibility of a hysterectomy.

The advantages of minimally invasive surgery are that it can be carried into the early postpartum period and might be used more frequently in the contemporary world. Successful laparoscopic coagulation/ligation of the bilateral uterine arteries/internal iliac arteries in the management of postpartum hemorrhage has also been reported in a few cases [12-15].

Our case illustrates a minimally invasive placenta extraction via laparoscopy, which results in less blood loss, quicker recovery times, and superior cosmetic outcomes compared to traditional obstetric methods.

## Conclusions

Placental retention requiring abdominal surgical intervention following vaginal delivery is a rare and unlikely situation. The main risk factors for this condition are uterine anomalies and preterm births. To our knowledge, this case is the first reported in the literature where laparoscopy was used to successfully remove the entrapped placenta, thus avoiding the need for laparotomy.
